# Cumulative small effect genetic markers and the detection of advanced colorectal neoplasias by population screening

**DOI:** 10.1186/1897-4287-10-S4-A26

**Published:** 2012-12-10

**Authors:** A Kurlapska, P Serrano-Fernández, T Starzyńska, E Małecka-Panas, G A Dąbrowski, T Dębniak, G Kurzawski, J Suchy, W Rogoza-Mateja, R J Scott, J Lubiński

**Affiliations:** 1 Department of Genetics and Pathomorphology of the Pomeranian Medical University, Szczecin, Poland; 2 Department of Gastroenterology, Pomeranian Medical University, Szczecin, Poland; 3 Department of Digestive Tract Diseases, Faculty of Medicine, Medical University of Łódź, Poland; 4 Department of Gastroenterology and Internal Medicine, Medical University of Białystok, Poland; 5 School of Biomedical Sciences and Pharmacy, University of Newcastle, Australia

## 

With the instigation of population screening strategies to reduce the burden of colorectal cancer, a cost effective approach remains an elusive goal. Genetic markers associated with colorectal cancer have the potential to be used for the early identification of patient groups at elevated risk of disease. The choice of genetic markers that can be used for screening purposes is population specific.

In this report we have genotyped 3059 individuals for 13 markers that have been associated with colorectal cancer risk. The participants underwent colonoscopy and controls with clear colonoscopy (1838) were compared to cases with advanced colorectal neoplasia (213). Logistic regression analysis, adjusted for sex and age at colonoscopy, showed that only one of the markers (rs4779584) was significantly associated with the risk of advanced colorectal neoplasia (OR = 1.93; 95% CI = 1.22 – 2.99; p-value = 0.004; sensitivity = 20%). A combination of 7 markers (rs4779584, rs2578187, rs3802842, rs6983267, NOD2 5020insC, rs4464148 and rs4939827) showed an optimal trade-off minimizing the number of markers considered, while maximizing the relative size of the carrier group and the risk associated to it (e.g. for at least 4 cumulated risk markers, OR = 4.23; 95% CI = 1.5 – 10.4; p-value = 0.0036; sensitivity = 4.7%). For this combination of 7 markers the linear cumulative risk model was statistically significant (p=7.0·10^-5^) after adjustment for sex and age at colonoscopy.

The identification of such cumulative models could be valuable in better defining a group of persons, within a given population, that are most likely to benefit from screening for colorectal cancer.

